# Variability of Coastal and Ocean Water Temperature in the Upper 700 m along the Western Iberian Peninsula from 1975 to 2006

**DOI:** 10.1371/journal.pone.0050666

**Published:** 2012-12-04

**Authors:** Fran Santos, Moncho Gómez-Gesteira, Maite deCastro, Inés Álvarez

**Affiliations:** 1 EPphysLab (Environmental Physics Laboratory), Universidade de Vigo, Ourense, Spain; 2 CESAM, Departamento de Física, Universidade de Aveiro, Aveiro, Portugal; Plymouth University, United Kingdom

## Abstract

Temperature is observed to have different trends at coastal and ocean locations along the western Iberian Peninsula from 1975 to 2006, which corresponds to the last warming period in the area under study. The analysis was carried out by means of the Simple Ocean Data Assimilation (SODA). Reanalysis data are available at monthly scale with a horizontal resolution of 0.5°×0.5° and a vertical resolution of 40 levels, which allows obtaining information beneath the sea surface. Only the first 21 vertical levels (from 5.0 m to 729.35 m) were considered here, since the most important changes in heat content observed for the world ocean during the last decades, correspond to the upper 700 m. Warming was observed to be considerably higher at ocean locations than at coastal ones. Ocean warming ranged from values on the order of 0.3°C dec^−1^ near surface to less than 0.1°C dec^−1^ at 500 m, while coastal warming showed values close to 0.2°C dec^−1^ near surface, decreasing rapidly below 0.1°C dec^−1^ for depths on the order of 50 m. The heat content anomaly for the upper 700 m, showed a sharp increase from coast (0.46 Wm^−2^) to ocean (1.59 Wm^−2^). The difference between coastal and ocean values was related to the presence of coastal upwelling, which partially inhibits the warming from surface of near shore water.

## Introduction

Numerous studies on global climate change have come to the conclusion that the last three decades of the twentieth century were the most intense warming period ever observed. The warming experienced by the world’s oceans over the last century was not uniform either in time or in space [1]–[5]. Several warming-cooling cycles have been detected over the 20^th^ century both at global and regional scales [6]–[10]. In addition, these changes were not evenly distributed around the world ´s oceans, with some regions warming faster or slower than the global average [11], [12]. In particular, the Atlantic Ocean is responsible for one third of the increase in heat content observed from 1955 to 1998 [13]–[15]. These differences are even more marked at regional scales where some areas warm at higher or lower rates than others due to changes in winds, ocean currents, thermohaline depth and upwelling [16]–[21]. Even recently, some regions have shown the existence of different surface warming rates at coastal and ocean locations [22], [23].

The variables most widely used to analyze the ocean warming have been the sea surface temperature (SST) and the ocean heat content. Both variables are complementary providing information about surface temperature trends and heat stored in the ocean between surface and a depth which typically ranges from 700 to 3000 m. In general, most of authors consider the upper 700 m since, according to [14], a great percentage of the warming is produced in the first 700 m while the layer 1000–3000 m provides only the 9% of the global increase. This picture was corroborated by further studies [24]–[26]. Only recently, some authors have claimed that there has been a significant heat content increase below 700 m during the last few years [27]. A clear comparison between heat content changes for the depths 0–700 and 700–2000 can be seen in [28]. SST is also a key parameter in the ocean-atmosphere heat exchange and hence in the climatic regulation. Numerous global and regional studies tried to quantify trends in SST showing a remarkable dependence on spatial and temporal scales [3], [5], [29]–[32].

To examine the role played by the ocean in climate change it is necessary to have an appropriate database in order to analyze the variables that can be used to describe the ocean variability. During the last decades, the most commonly used ocean database has been the World Ocean Database [14]. During the last decades, a great effort has been also devoted to develop reliable SST series with global coverage, first by means of measurements from voluntary observation ships, drifters and moored buoys [33], [34] and then by means of satellite-derived data. Great effort has also been devoted to correct uncertainties in the SST data (for a complete understanding of the different biases see [35]–[41]). More recently, some data assimilation projects like Simple Ocean Data Assimilation (SODA) have reanalyzed data from different sources (oceanographic cruises, satellite, model simulations). These data assimilation projects provide valuable information beneath the sea surface. In this way, it is possible to obtain a complete view of the different- scale hydrographic processes by analyzing ocean patterns both at global [42], [43] and basin scale [44]–[46].

The aim of this study is to describe the differences in the variability of temperature and heat content between coastal and ocean locations along the western Iberian Peninsula from 1975 to 2006. This analysis will be carried out by means of data obtained from SODA reanalysis. As far as we know, only SST patterns had been considered in the study area. In the present study, information about the vertical structure of temperature changes will also be provided.

## Results

**Figure 1:**
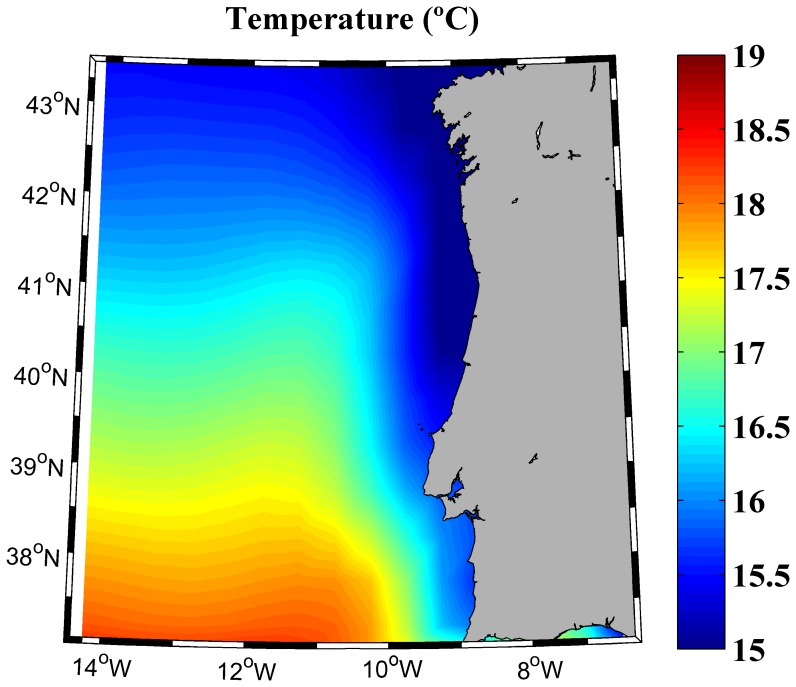
Near surface water temperature (°C) averaged for the mixed layer (from 5.0 to 46.6 m) over the period 1975–2006.

The area located in front of the Western Iberian Peninsula (from 37.25 to 43.25°N and from 9.75 to 14.75°W) has been considered. This region is characterized by a shoreline perpendicular to the equator, being coastal upwelling the main driving mechanism (see [10] and the references herein). The chosen period, 1975–2006, corresponds to the last warming period both in the North Atlantic and in the area under study [9], [10], [20], [22]. Near surface water temperature averaged for the mixed layer (5.0 to 46.6 m) from 1975 to 2006 is shown in Figure 1. The criterion followed to calculate the mixed layer is described in *Data and Methods* section. Costal water is observed to be considerably colder than ocean water at the same latitude, showing the imprint of coastal upwelling.

**Figure 2:**
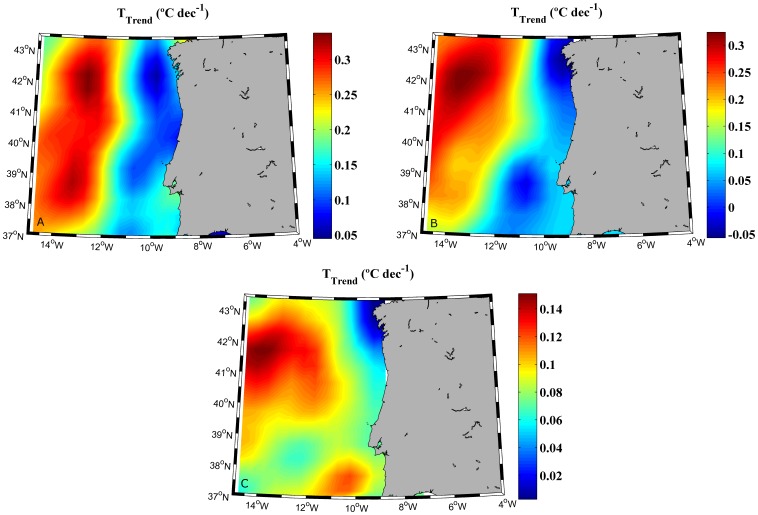
Horizontal map of the temperature trend (°C dec^−1^) calculated at different depths over the period 1975–2006: A mixed layer (from 0 to 46.6 m deep), B thermocline base (from 58 to 197 m deep) and C intermediate water (from 229.5 to 729.4 m deep).

Figure 2 shows the temperature trend calculated at different depths over the period 1975–2006. Figure 2A shows the temperature trend for the mixed layer. The warming trend observed near coast (∼0.10–0.15°C dec^−1^), is considerably lower than in the ocean (0.25–0.35°C dec^−1^). Temperature trends were also calculated for the thermocline base (Figure 2B), which ranges approximately from 50 to 200 m. There, coastal temperature trends are on the order of 0–0.1°C dec^−1^ and ocean trends on the order of 0.15–0.25°C dec^−1^. Finally, trends were also calculated for the intermediate water which ranges approximately from 200 to 700 m (Figure 2C). The pattern observed from 43.25°N to approximately 38.25°N is similar to the previous ones. Costal trends on the order of 0–0.05°C dec^−1^ and ocean trends on the order of 0.10–0.15°C dec^−1^ can be observed for these latitudes. This pattern is not observed further South due to the input of Mediterranean Water. Due to the identifiable presence of this water mass in the southernmost part of the study area, only the region ranging from 38.25 to 43.25°N will be considered from now on.

**Figure 3:**
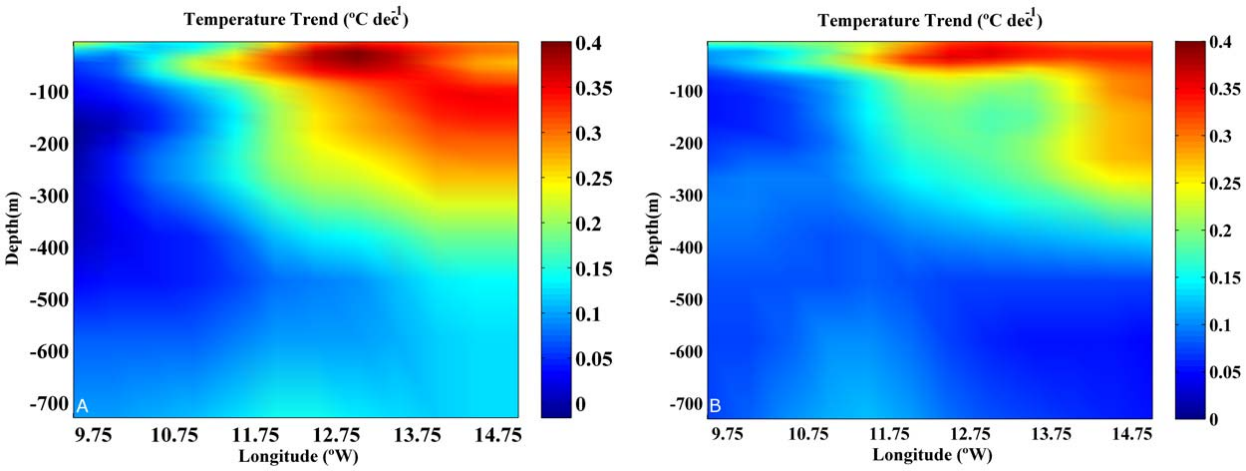
Temperature trend (°C dec^−1^) for the period 1975–2006 calculated at two latitudes: A 41.75°N and B 39.75°N.

Vertical profiles of temperature trends were used to analyze the water column (Figure 3). Profiles were calculated at two different latitudes 41.75 °N (Figure 3A) and 39.75 °N (Figure 3B). Some common features can be observed in both plots. The ocean warming (10.75 to 14.75°W) is considerably higher than the coastal one (9.75 to 10.75°W). The highest warming (0.2–0.4°C dec^−1^), is found at ocean locations covering the upper 300 m. On the contrary, the warming hardly attains 0.2°C dec^−1^ near shore and only affects the upper 20 m, being practically negligible (<0.1°C dec^−1^) below 50 m. These common features can also be observed at the rest of latitudes from 38.25 to 43.25°N (not shown).

**Figure 4:**
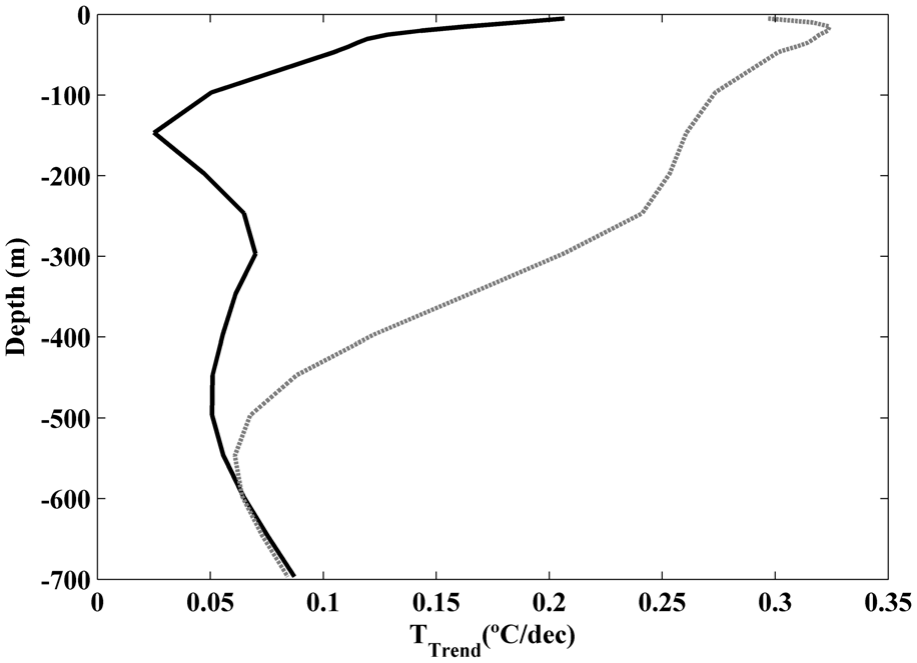
Vertical profiles of temperature trends (°C dec^−1^) meridionally averaged for the three longitudes nearest to the coast (solid line) and for the three longitudes farthest the coast (dashed line).

The vertical profile of coastal temperature trends (Figure 4, solid line) was calculated by averaging the temperature trends considering the three longitudes closest to coast and all latitudes from 38.25 to 43.25°N. The coastal vertical profile allows characterizing trends in the zone influenced by upwelling processes. Previous studies carried out on the Western Iberian Peninsula [22] have shown that zone influenced by coastal upwelling is mainly constrained to the first degree from coast. The ocean profile (Figure 4, dashed line) was calculated in the same way but considering the three longitudes farthest from coast. In this way, the oceanic trend is not influenced by coastal processes. The ocean warming is higher than the coastal one as mentioned in Figure 3. In particular, values on the order of 0.3°C dec^−1^ are observed near surface at the ocean decreasing with depth till reaching values below 0.1°C dec^−1^ at 500 m. The coastal warming is much smaller, reaching values close to 0.2°C dec^−1^ near surface and decreasing rapidly at values below 0.1°C dec^−1^ for depths on the order of 50 m.

**Figure 5:**
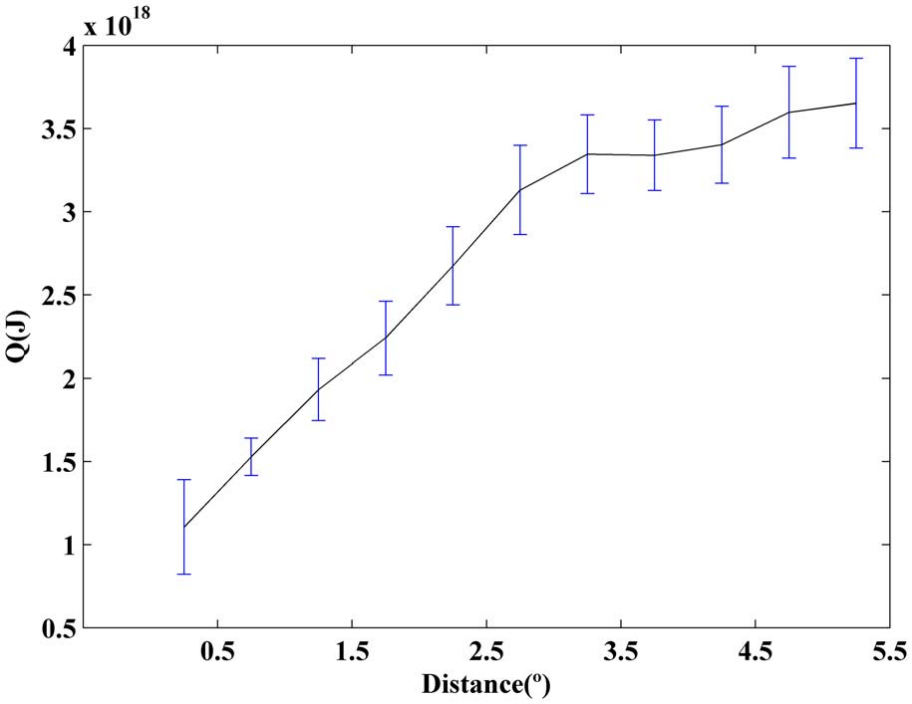
Heat content anomaly, Q (J), meridionally averaged from 38.25 to 43.25°N over the period 1975–2006. Error bars were calculated using the standard deviation of monthly data, σ (Q), divided by the square root of the number of data. All trends are significant with p<0.05.

Once the difference between coastal and ocean warming has been analyzed, we will quantify the changes observed in heat anomaly (Q), which was calculated from temperature anomaly following Eq. 1 in *Data and Methods* section. The heat content anomaly was meridionally averaged from 38.25 to 43.25°N over the period 1975–2006 (Figure 5). Q shows a sharp increase from coast, 1.1×10^18 ^J (0.46 Wm^−2^), to ocean, 3.7×10^18 ^J (1.59 Wm^−2^). Error bars were calculated using the standard deviation of the monthly data, σ(Q), divided by the square root of the number of data.

**Figure 6:**
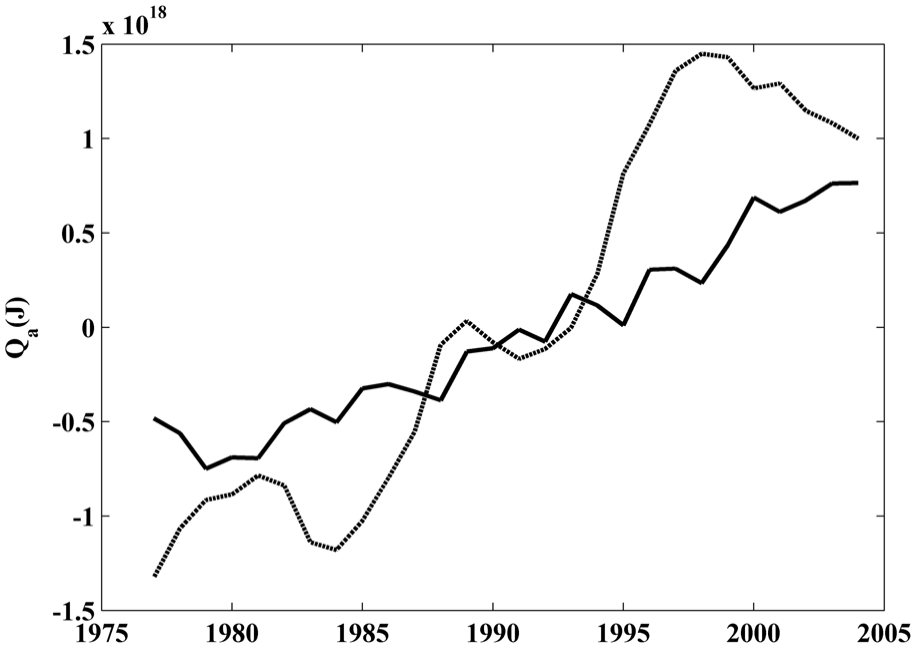
Time evolution of the heat content anomaly (Q (J)) meridionally averaged from 38.25 to 43.25°N over the period 1975–2006. The black solid line is the mean of the three nearest locations to the coast and the grey dashed line is the mean of the three most oceanward locations. A running average of ±2 years was considered to smooth the signal.

The time evolution of heat anomaly (Figure 6) is also different for coastal (solid line) and ocean (dashed line) locations. Coastal (ocean) heat anomaly was calculated by averaging heat anomaly at the three most landward (seaward) points. A running average (±2 years) was applied to smooth the signal. Heat content anomaly shows a sharp increase (∼1.1×10^18^J dec^−1^) at ocean locations and a smoother one (∼0.6×10^18^J dec^−1^) at coastal locations.

## Discussion

The difference in SST warming rates between near coast and at ocean locations had been previously described for the Western Iberian Peninsula [22] and for other areas like the Canary Upwelling System [47] and the Benguela Upwelling System [23]. Nevertheless, the extent of this difference with depth had not been analyzed in detail due to the lack of data along the water column. The use of a tridimensional data obtained from SODA has allowed a more in-depth analysis over the period 1975–2006. The warming trend (Figure 2A) observed for the mixed layer (from 0 to 46 m deep) near coast is considerably lower (∼0.10–0.15°C dec^−1^) than at the ocean (0.25–0.35°C dec^−1^). These values are similar to SST trends observed for the same area near shore (∼0.15°C dec^−1^) over the period 1985–2005 [20]. Similar SST trends (0.28°C dec^−1^ near shore and 0.30°C dec^−1^ at open sea locations) were found over the period 1974–2008 [22]. Finally, a similar warming trend was measured near coast (0.24°C dec^−1^) over the period 1974–2007 for the NW corner of the Iberian Peninsula [10]. In spite of slight differences in the observed trends, which are mostly due to differences among databases and to the extent of the areas and periods under study, these previous studies highlight the existence of different SST warming rates at coastal and ocean locations. However, little was known about temperature changes along the water column. Figure 2B shows that the differences between coast and ocean previously described for the mixed layer are kept for the thermocline base (from 58 to 197 m deep). This situation is not maintained for the whole area when analyzing intermediate water (from 229.5 to 729.4 m deep) as it can be observed in Figure 2C. At these depths the warming pattern changes south of 38.25°N due to the intrusion of the Mediterranean Water, which flows approximately between 400 and 1500 m (for a brief summary about the different water masses in the zone the reader is referred to [48], [49] and to the references therein).

The meridional average of trends was calculated for coastal and ocean locations considering all latitudes ranging from 38.25 to 43.25°N (Figure 4). The coastal vertical profile shows a slight warming (∼0.2°C dec^−1^) near surface (upper 20 m), which decreases rapidly and becomes negligible below 50 m. On the contrary, the ocean vertical profile shows a stronger surface warming (0.2–0.4°C dec^−1^), which penetrates down to 300 m. That ocean vertical profile of temperature trend is similar to the warming decrease with depth described for the Bay of Biscay along the upper 400 m [50] using the World Ocean Database. There, the authors obtained a maximum trend (∼0.25°C dec^−1^) at subsurface layer, which is similar to the one observed in the present study (∼0.32°C dec^−1^) for ocean locations. In addition, their trend does not decrease abruptly during the first tens of meters but it remains positive till approximately 500 m. The same was observed in our study, where trends on the order of 0.05°C dec^−1^ are observed at 600 m. Note that study described in [50] averages the whole Bay of Biscay, in such a way that the mean values clearly resemble the oceanic behavior since the coastal points are only a small percentage of the total set.

It should be noted that the area has suffered a noticeable warming from 1975 on. Actually, according to [10], the land temperature in the area has increased at an approximate rate of 0.5°C dec^−1^. At ocean locations, the upper layers of the ocean are also affected by the warming observed for air temperature. There, heat is mainly transported down from surface to lower layers by thermal diffusion. Near shore, coastal upwelling is the most important forcing mechanism, in such a way that cold water is pumped intermittently from below to near surface layers. Thus, diffusion is offset by advection, which mixes deeper colder water with warmer surface water. Thus, diffusive atmospheric heating is constrained to near surface layers, resulting in a weaker coastal warming. This difference in coastal and ocean warming rates is not necessarily correlated with changes in upwelling intensity. In fact, the mechanism proposed in [51] by which global warming would result in increased upwelling intensity is the subject of wide controversy in the area under study [17], [22], [52]–[54]. Even some authors found that upwelling intensification coincided with warmer SST in the California Current System, proving that other factors like long-term SST warming can mask the cooling effect associated to upwelling [55]. Thus, the mere presence of upwelling may be sufficient to constrain the warming to the upper layers. However, the difference between coastal and ocean trends are more marked in those regions that have experienced changes in upwelling intensity during the last decades as shown in recent studies carried out in the Benguela Upwelling System [23] and the Canary Upwelling System [47].

The heat content anomaly meridionally averaged from 38.25 to 43.25°N increases from coast (0.46 Wm^−2^) to ocean (1.59 Wm^−2^). The increment for the ocean is considerably higher than that obtained for the world ocean (0.20 Wm^−2^) and for the Atlantic Ocean (0.52 Wm^−2^) [14]. Note that, the period considered in that study was 1955–1998, which contains a cooling part from the mid- fifties to the mid- seventies and a warming part from the mid-seventies on. Later, these results were corrected increasing to 0.25 Wm^−2^ for the world ocean over the period 1969–2008 considering the upper 700 m [24]. More recently, an increase of 0.64 Wm^−2^ was detected for the world ocean over the period 1993–2008 considering the upper 700 m [26]. In addition, an increase on the order of 0.8 Wm^−2^ can be estimated for the upper North Atlantic (0–700 m) over the period 1968–2003 [14]. Even, more recently, [28] calculated the ocean heat content over the period 1955–2010. Values of 0.27 Wm^−2^ (0.39 Wm^−2^) were obtained for the world ocean considering the upper 700 and 2000 m respectively. In the particular case of the Atlantic Ocean, the increase is on the order of 0.54 Wm^−2^ (0.81 Wm^−2^) for the upper 700 m (2000 m). Finally, different studies shows that the heat content anomaly calculated using SODA for the band 15°–60° N over the 1961–2001 period [42] is about 50% higher than obtained by [14] for the same years and band. Even, considering that there are significant differences among heat content anomalies calculated using different databases, the present study shows that the rate between heat content increase at coastal and ocean locations along the WIP is on the order of one third. This is in good agreement with previous studies carried out by the authors in different upwelling areas [22], [23], [47], which show that SST warming at coastal locations is less intense than at adjacent ocean locations.

## Data and Methods

Sea Temperature data were obtained from the Simple Ocean Data Assimilation (SODA). Reanalysis data covering the period 1958–2008 are available at monthly scale with a horizontal resolution of 0.5°×0.5° and a vertical resolution of 40 levels (http://www.atmos.umd.edu/~ocean/). For detailed information about the methodology the reader is referred to [56], [57]. Only the first 21 vertical levels of the SODA database (from 5.0 m to 729.35 m) were considered in the present study since, according to [14], [24], the most important changes in heat content observed for the world ocean during the last decades, correspond to the upper 700 m. Trends were calculated over the period 1975 to 2006, which corresponds to the last warming cycle in the North Atlantic.

Other data bases were considered for comparison purposes. Thus, daily sea surface temperature (SST) data were obtained from daytime measurements carried out by the Advanced Very-High Resolution Radiometer (AVHRR) onboard of NOAA series satellites. Data were retrieved from Pathfinder Version 5.2 (ftp://ftp.nodc.noaa.gov/pub/data.nodc/pathfinder/Version5.2/) with an approximate spatial resolution of 4×4 km extending from 1982 to nowadays. Data treatment is described in [47]. SST data were also obtained from the UK Meteorological office, Hadley Center HadISST1.1- Global sea- Ice coverage and SST (http://badc.nerc.ac.uk/data/hadisst) [58]. Data are available from 1870 on, with monthly periodicity on a 1°×1° grid with global coverage.

The mean temperature provided by SODA at surface layer (upper 5 m, Figure S1A) can be compared to the mean of SST obtained from Pathfinder (Figure S1B) and HadISST (Figure S1C). Macroscopically, similar qualitative and quantitative behaviour can be observed in the three frames corresponding to the period 1982–2006, which is common to the three databases. Actually, the biggest differences are observed between HADISST and the rest of the databases at coastal areas due to its coarser grid.

The comparison among temperature trends (T_trend_) was calculated for a transect located at 41.75°N (Figure S1D). Sea temperature data were first re-meshed on the same spatial scale (0.5° ×0.5°) and calculated for the same period (1982–2006). The trend provided by the three databases is always positive and on the same order of magnitude. The trend was also observed to decrease landward in the three cases.

Following [50], three different layers will be considered in the further analysis for the ocean vertical profile of SODA: the mixed layer (from 5.0 to 46.6 m deep), the thermocline base (from 58 to 197 m) and the intermediate water (from 229.5 to 729.4 m). The mixed layer depth (MLD) was calculated following the temperature criterion, SST- ST(z) = 0.2°C, described in Table 1 in [59], where ST(z) is the temperature at depth (z). This criterion indicates that the layer depth is defined as the depth where the temperature is 0.2°C less that the SST. The mean annual mixed layer depth was averaged over the period 1975–2006 for two transects perpendicular to the shore line (transect 1 at 41.75°N and transect 2 at 39.75°N, see Figure S2). The mean MLD obtained for both transect is −44.5 m and −43.5 m respectively, in good agreement with the value shown above. Note that the layers in SODA are not equally spaced and the closest ones are located at −35.8, −46.6 and −58.0 m respectively.

Heat content anomaly was calculated at each grid point over the period 1975–2006 in terms of temperature anomaly, following the expression:
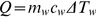
(1)where *c_w_* = 3.981×10^3^ J (°C-kg) ^−1^ is the specific heat of seawater at constant pressure at the sea surface, m_w_ = ρ_w_V_w_ is the mass of the seawater block, where *ρ_w_* = 1.02767×10^3 ^kg/m^3^ is the density and V_w_ = (0.5×111120)(0.5×111120 *cos*((2π/180) *lat*)) ×*Δh* the volume, being *lat* the latitude of the water parcel and *Δh* the water column height from surface to 729.35 m. *c_w_* and *ρ_w_* values were taken from [60]. Finally, temperature anomaly was calculated following ΔT_w_ = dT*Δ*t (°C), where dT is the temperature trend (°C/year) and *Δ*t the period under study (in years).

## Supporting Information

Figure S1 (A) Mean sea temperature (°C) at surface layer (upper 5 m) from the Simple Ocean Data Assimilation (SODA, http://www.atmos.umd.edu/~ocean/), (B) mean SST from Pathfinder (ftp://ftp.nodc.noaa.gov/pub/data.nodc/pathfinder/Version5.2/) and (C) mean SST from HadISST1.1- Global sea- Ice coverage and SST (http://badc.nerc.ac.uk/data/hadisst). Data were averaged over the period 1982–2006. (D) SST trend (°C dec-1) calculated at 41.75°N from 1982 to 2006 using the three data bases mentioned above. SST data were previously re-meshed to a common spatial resolution of 0.5° ×0.5°. Legend in the figure P: Pathfinder; S: SODA; H: HasISST.
(TIF)

Figure S2 Mixed layer depth (MLD) at two transects located at 41.75°N (black line) and 39.75°N (gray line). The MLD was calculated following the temperature criterion SST -ST(z) = 0.2°C, where ST(z) is the water temperature at depth (z) [59]. This criterion identifies the MLD as the depth at which the temperature is 0.2°C lower than the SST.
(TIF)
