# Supplementary figures and images for: Variability of Coastal and Ocean Water Temperature in the Upper 700 m along the Western Iberian Peninsula from 1975 to 2006
Source: PLoS One. 2012 Dec 4;7(12):e50666. doi: 10.1371/journal.pone.0050666 (PMC3514266; doi:10.1371/journal.pone.0050666)

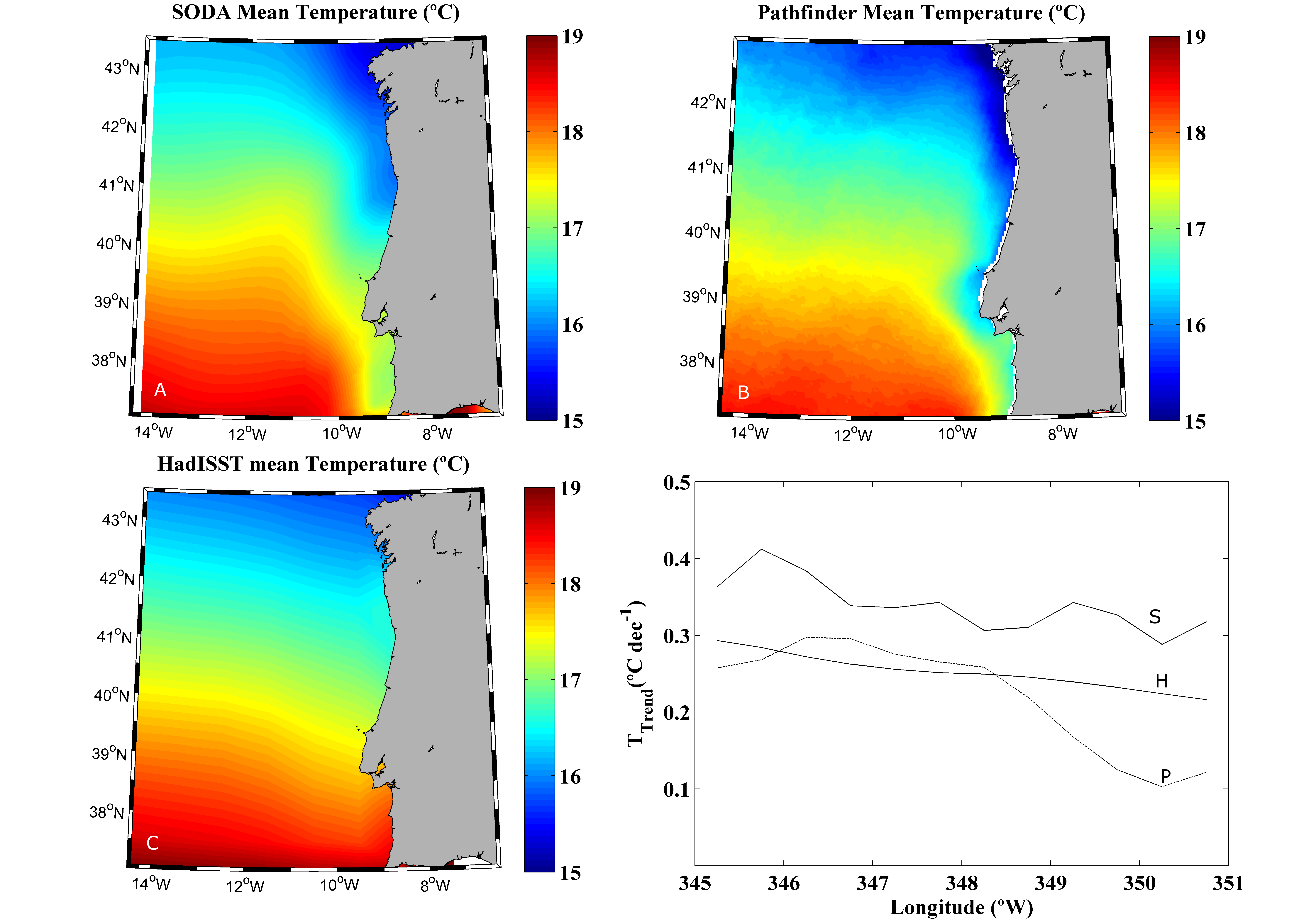

Supplement: Figure S1 — (A) Mean sea temperature (°C) at surface layer (upper 5 m) from the Simple Ocean Data Assimilation (SODA, http://www.atmos.umd.edu/~ocean/), (B) mean SST from Pathfinder (ftp://ftp.nodc.noaa.gov/pub/data.nodc/pathfinder/Version5.2/) and (C) mean SST from HadISST1.1- Global sea- Ice coverage and SST (http://badc.nerc.ac.uk/data/hadisst). Data were averaged over the period 1982–2006. (D) SST trend (°C dec-1) calculated at 41.75°N from 1982 to 2006 using the three data bases mentioned above. SST data were previously re-meshed to a common spatial resolution of 0.5° ×0.5°. Legend in the figure P: Pathfinder; S: SODA; H: HasISST. (TIF) [file pone.0050666.s001.tif]

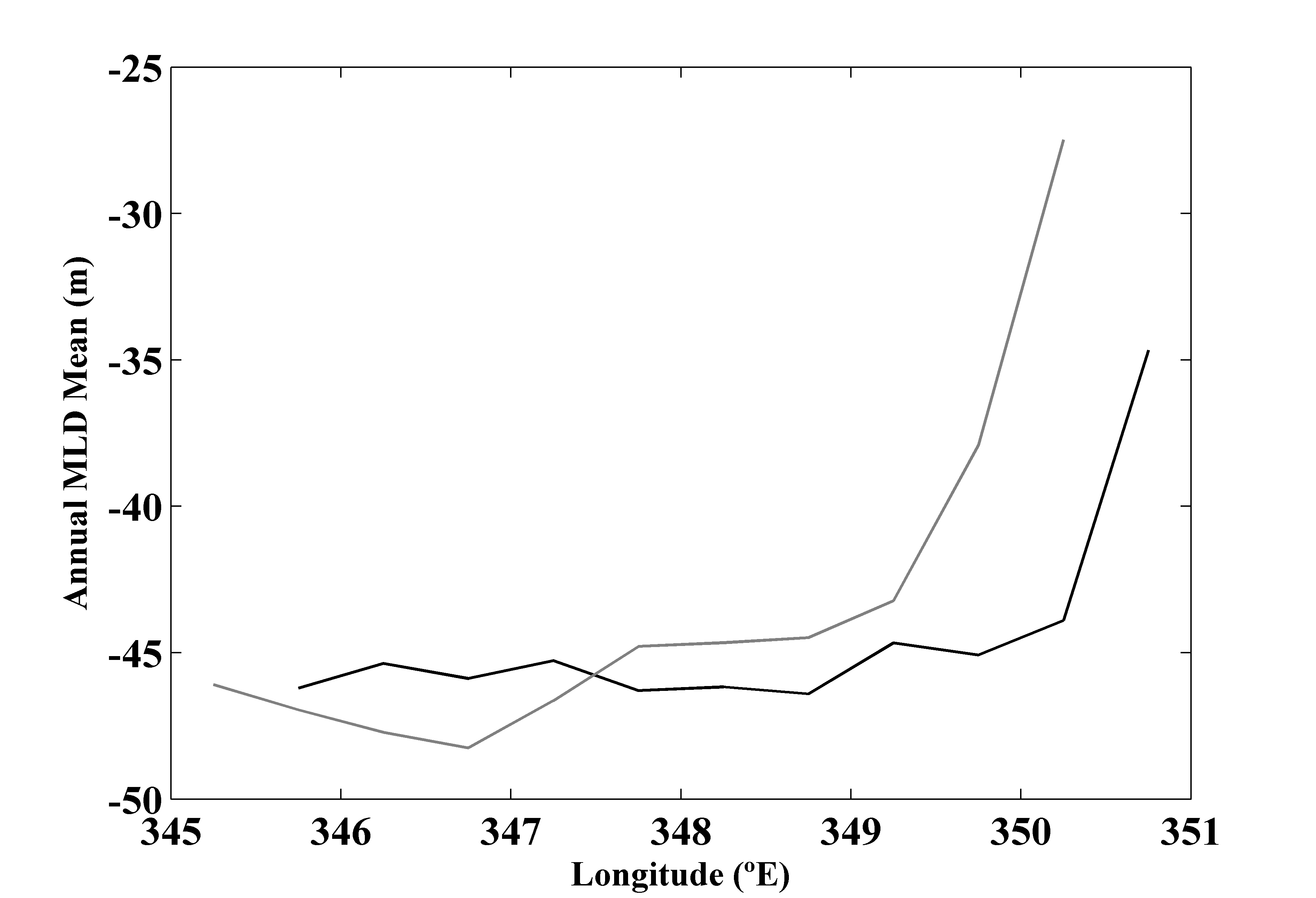

Supplement: Figure S2 — Mixed layer depth (MLD) at two transects located at 41.75°N (black line) and 39.75°N (gray line). The MLD was calculated following the temperature criterion SST -ST(z) = 0.2°C, where ST(z) is the water temperature at depth (z) [59]. This criterion identifies the MLD as the depth at which the temperature is 0.2°C lower than the SST. (TIF) [file pone.0050666.s002.tif]
